# Natural History and the Burden of Malaria During the First Year of Life in the High-Transmission Setting of Uganda

**DOI:** 10.4269/ajtmh.25-0695

**Published:** 2026-04-02

**Authors:** Miriam Aguti, Joaniter I. Nankabirwa, Jimmy Kizza, Abel Kakuru, Timothy Ssemukuye, Harriet Adrama, Peter Olwoch, Bishop Opira, Baker Odongo, Kylie Camanag, Miriam Nakalembe, Tamara Clark, Philip J. Rosenthal, Grant Dorsey, Prasanna Jaganathan, Moses R. Kamya

**Affiliations:** ^1^ Infectious Diseases Research Collaboration, Kampala, Uganda;; ^2^ School of Medicine, Makerere University College of Health Sciences, Kampala, Uganda;; ^3^ Department of Community and Public Health, Busitema University, Tororo, Uganda;; ^4^ Department of Medicine, Stanford University School of Medicine, Stanford, California, USA;; ^5^ Department of Medicine, University of California, San Francisco, California, USA

## Abstract

Infants under 1 year of age are considered partially protected from malaria because of maternal antibodies and fetal hemoglobin. However, emerging evidence suggests that the malaria burden in this age group may be underestimated. A cohort of 855 infants in Busia District, Uganda, was enrolled in the present study to characterize malaria incidence and parasite prevalence during the first year of life and to identify risk factors for these outcomes. The study was conducted from 2021 to 2025, before the malaria vaccine roll-out. Infants born to HIV-uninfected women were enrolled at 4–8 weeks of age and followed by active and passive case detection to 1 year of age in a dedicated study clinic that is open 7 days/week. Routine visits every 4 weeks included assessments for parasitemia via microscopy and quantitative polymerase chain reaction testing. Over 706.7 person-years of follow-up, 662 malaria episodes occurred. The overall prevalence of microscopic parasitemia was 7.9%, and the combined prevalence of microscopic and submicroscopic parasitemia was 21.8%. Sickle cell trait (Hemoglobin AS) conferred 39% protection against symptomatic malaria but was not associated with the risk of parasitemia. Modern housing construction and higher maternal education were independently associated with reduced malaria risk. District-wide distribution of long-lasting insecticide-treated nets containing alpha–cypermethrin plus chlorfenapyr in October 2023 was followed by an 80% reduction in malaria incidence and significant declines in parasitemia prevalence. These findings underscore the urgent need for age-appropriate preventative interventions targeting young infants, such as earlier vaccine administration or treatment with monoclonal antibodies, alongside sustained investment in next-generation vector control and attention to socioeconomic determinants of malaria risk.

## INTRODUCTION

Malaria remains a leading public health concern, particularly in sub-Saharan Africa, which is responsible for more than 95% of global malaria morbidity and mortality.^1^ Children under 5 years of age are disproportionately affected by malaria, and infants under 1 year of age represent a vulnerable group that is often understudied.^2^^,^^3^ Although infants younger than 6 months of age are often considered partially protected from malaria because of the presence of maternal antibodies^4^ and fetal hemoglobin,^5^^,^^6^ emerging evidence suggests that the burden of malaria in this age group may be substantially underestimated.^2^^,^^7^^,^^8^ Indeed, evidence indicates that infants as young as 1 to 2 months of age can experience clinical malaria, with the malaria risk increasing substantially after 2 months of age.^2^^,^^7^^,^^8^

Malaria prevention strategies targeting infants under 1 year of age are limited, especially in regions with perennial malaria transmission. In such settings, in addition to long-lasting insecticide-treated nets (LLINs), the WHO recommends perennial malaria chemoprevention (PMC). Perennial malaria chemoprevention with sulfadoxine–pyrimethamine (SP) following the immunization schedule of the expanded program on immunizations (formerly referred to Intermittent Preventive Treatment of malaria in Infants-IPTi) has been shown to be safe and effective against malaria during the first year of life;^9^ however, uptake has been low, in part because it provides a relatively low level of protection against symptomatic malaria^9^ and its efficacy is limited by growing SP resistance.^10^ Recently, two malaria vaccines, RTS,S and R21, have been approved and are beginning to be deployed across Africa, including Uganda, among children 5–17 months of age. However, in previous studies, optimal malaria vaccine efficacy was achieved only after three doses (administered at 8–9 months of age), leaving a protection gap for younger infants. Finally, there are no tailored WHO malaria treatment guidelines for infants under 6 months of age. The lack of targeted malaria prevention and treatment strategies in very young children, along with the new evidence suggestive of a higher disease burden than previously anticipated in this age group, highlights an urgent need for research to guide evidence-based policy and clinical practice.

To address these gaps, a prospective cohort study was conducted to characterize the natural history and burden of malaria during the first year of life in a high-transmission area of Uganda. The aim was to inform the development of effective, age-appropriate interventions and treatment guidelines for infants living in perennial, high-transmission settings.

## MATERIALS AND METHODS

### Study participants and setting.

Infants followed in this cohort study were born to HIV-uninfected women who participated in a double-blind, randomized controlled trial of three different intermittent preventive treatment in pregnancy regimens (Dihydro-artemisinin Piperaquine-Sulfadoxine Pyrimethamine [DPSP] study, NCT04336189).^11^ Briefly, 2,757 pregnant women were randomly assigned in a 1:1:1 ratio to receive monthly intermittent preventative treatment of malaria in pregnancy (IPTp) with either SP, dihydroartemisinin–piperaquine (DP), or a combination of DP and SP, and followed to delivery. Participants were approached at their 4-week postpartum visit and asked whether they would like to enroll their infants in the present follow-up study. Infants were screened and enrolled if they were between 4 and 8 weeks of age, born to mothers who participated in the DPSP trial, lived in Busia District, and had parents or guardians who provided written informed consent and agreed to attend a dedicated study clinic for all routine medical care and to avoid medications administered outside the study clinic. Consecutive enrollment continued until the desired sample size of 871 infants was achieved.

The present study was conducted between November 2021 and January 2025 in Busia District, southeastern Uganda. Busia is a high-malaria-endemic district with perennial transmission, marked by two seasonal peaks. Before 2013, vector control in Busia had been limited to the targeted distribution of LLINs through antenatal care services. Universal distribution campaigns of free LLINs were conducted in Busia District in May 2013, May 2017, December 2020, and October 2023. The first two campaigns involved standard pyrethroid LLINs, the 2020 campaign involved LLINs containing deltamethrin plus piperonyl butoxide (PBO; PermaNet 3.0 [Vestergaard Sarl/Frandsen, Lausanne, Switzerland]), and the 2023 campaign involved LLINs containing alpha–cypermethrin plus chlorfenapyr (Interceptor G2 [BASF SE, Ludwigshafen, Germany]). Indoor residual spraying of insecticides has never been implemented in Busia District.

### Study procedures.

At enrollment, a baseline evaluation was conducted on all infants, including a detailed medical history, a focused physical examination, and blood collection via venipuncture for thick blood smear testing, quantitative polymerase chain reaction (qPCR) testing, hemoglobin measurement, sickle cell testing, and storage for future molecular studies. Additionally, all infants received an LLIN containing deltamethrin plus PBO (PermaNet 3.0) at enrollment. Participants with sickle cell disease were given monthly chemoprophylaxis with SP. Study participants were encouraged to visit a dedicated study clinic, open 7 days per week, for all their medical care and provided with transportation reimbursement. Routine visits were conducted every 4 weeks and included a standardized evaluation and collection of blood via finger prick for thick blood smear testing, qPCR testing, hemoglobin measurement (at 8, 24, and 52 weeks), and storage for future studies. Study participants with a fever (tympanic temperature* >*38.0°C) or a history of fever in the previous 24 hours at the time of any clinic visit had a thick blood smear read immediately. If the thick blood smear result was positive on light microscopy, the patient was diagnosed with symptomatic malaria, which was managed in accordance with national guidelines.^12^ Study participants who missed their scheduled routine visits were visited at home and requested to visit the study clinic as soon as possible. All enrolled participants were followed until they reached 1 year of age, unless they were prematurely withdrawn. Participants were withdrawn if they 1) moved out of the cohort household, 2) were unable to be located for* >*3 months, 3) had their informed consent withdrawn, or 4) were unable to comply with the study schedule and procedures.

Study procedures for the mothers have been previously described.^11^ Briefly, after enrollment, women were visited at home, where a household survey was conducted to collect detailed information about the participants’ households using a structured questionnaire. Data from this household survey were also applied to infants enrolled in the present study.

### Laboratory procedures.

Blood smears were stained with 2% Giemsa and read by experienced microscopists. A blood smear result was considered negative when the examination of 100 high-power fields did not reveal asexual parasites. For quality control, all slides were read by a second microscopist, and a third reviewer settled any discrepant readings. Blood samples collected at enrollment and at each routine visit were tested for the presence of submicroscopic parasitemia using a highly sensitive qPCR assay targeting the multicopy conserved *var* gene acidic terminal sequence with a lower limit of detection of 0.05 parasites/*µ*L.^13^ Sickle cell status was ascertained by testing samples using hemoglobin electrophoresis at the Ugandan Central Public Health Laboratory.

## STATISTICAL ANALYSES

Data were collected using standardized case record forms and entered into Microsoft Access (Microsoft Corp., Redmond, WA). All statistical analyses were performed using Stata version 19 (StataCorp LLC, College Station, TX).

Exposure variables of interest included maternal characteristics (gravidity, level of education, and IPTp regimen received during pregnancy), infant characteristics (sex, sickle hemoglobin genotype, and whether the child was born small for gestational age [SGA]), the date the observation period began (before [November 2021–May 2023], during [June 2023–October 2023], or after [November 2023–March 2024] the most recent universal LLIN distribution campaign), and household characteristics (household wealth and home construction). Principal component analysis was used to generate a wealth index on the basis of ownership of common household items. Households were ranked by wealth scores and grouped into tertiles to provide a categorical measure of socioeconomic status. House types were classified on the basis of definitions previously developed for the study area.^14^ Modern houses were defined as having plaster or cement walls, metal or wooden roofs, and closed eaves; all other houses were defined as traditional.

Three outcome measures were assessed: 1) the incidence of symptomatic malaria, with an incident episode defined as the presence of fever (history of fever in the past 24 hours or a tympanic temperature ≥38.0°C) with a positive thick blood smear not preceded by another malaria episode in the last 14 days; 2) the prevalence of microscopic parasitemia, defined as the proportion of routine samples positive for asexual parasites on microscopy; and 3) the prevalence of microscopic or submicroscopic parasitemia, defined as the proportion of routine samples positive for parasites on either microscopy or qPCR.

For all analyses, a modified intention-to-treat approach was used, including all children enrolled and followed through at least 12 weeks of age, with no missing sickle cell status or household survey information. Time at risk was calculated from 8 weeks of age and ended when the study participants reached 12 months of age or early study termination. Incident outcomes were compared using negative binomial regression models. Repeated prevalence measures were compared using log-binomial models with robust standard errors and generalized estimating equations to account for repeated measures in the same participant. Multivariate analyses included all exposure variables of interest assessed using univariate analyses (saturated models). In all analyses, two-tailed *P*-values <0.05 were considered statistically significant.

## RESULTS

### Characteristics of study participants.

**Figure 1.**
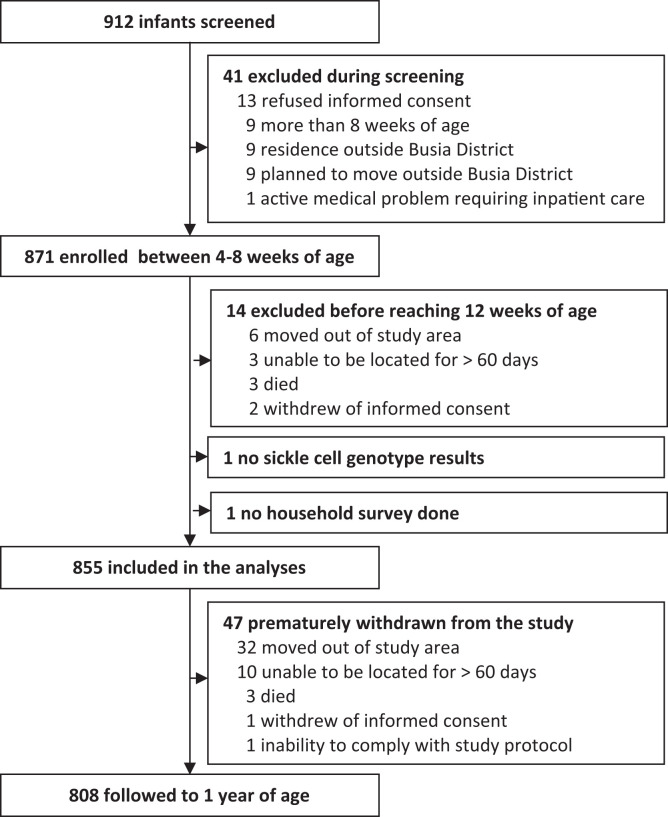
Study profiles.

Between November 2021 and March 2024, 912 infants were screened for eligibility, and 871 were enrolled in the present study (Figure 1). Of the total enrolled participants, 855 were included in the final analysis after excluding 14 infants who were withdrawn before reaching 12 weeks of age, one who lacked sickle cell genotype results, and one who did not have a completed household survey. Among those analyzed, 808 participants (94.5%) successfully completed follow-up through 12 months of age. A total of 44 participants were withdrawn prematurely, with the most common reason for withdrawal being relocation outside the study area. An additional three participants died, resulting in a total of 47 participants who did not successfully complete the study at 1 year of age.

Of the 855 infants included in the analyses, 23.2% were born to primigravid mothers, 69.6% were born to mothers with either primary education or no formal education, and 70.2% resided in traditional houses (Table 1). Overall, 48.4% were female, 21.2% had sickle cell trait, 0.7% had sickle cell disease, and 21.4% were born SGA. The date the observation period began (8 weeks of age) occurred after universal LLIN distribution (October 2023) in 38.0% of infants (Table 1).

**Table 1:** Risk factors for the incidence of symptomatic malaria between 8 weeks and 1 year of age

Risk Factor	Category (number of infants)	Episodes of Malaria (incidence PPY)	IRR (95% CI)	*P*-Value	aIRR* (95% CI)	*P*-Value
Maternal and household characteristics						
Gravidity	Multigravid (657)	500 (0.91)	Reference group		Reference group	
	Primigravid (198)	162 (1.02)	1.11 (0.83–1.47)	0.48	1.14 (0.88–1.47)	0.34
Maternal education	Secondary or higher (260)	167 (0.78)	Reference group		Reference group	
	None or primary (595)	495 (1.00)	1.29 (0.99–1.68)	0.06	1.34 (1.05–1.71)	0.02
Type of house construction	Modern (255)	135 (0.65)	Reference group		Reference group	
	Traditional (600)	527 (1.06)	1.64 (1.24–2.16)	<0.001	1.60 (1.23–2.08)	<0.001
Household wealth categories	Least poor (275)	172 (0.76)	Reference group		Reference group	
	Middle (298)	240 (0.97)	1.28 (0.95–1.73)	0.10	1.12 (0.85–1.48)	0.42
	Poorest (282)	250 (1.07)	1.41 (1.04–1.91)	0.03	1.04 (0.78–1.37)	0.81
Maternal IPTp regimen	SP (291)	230 (0.96)	Reference group		Reference group	
	DP (290)	235 (0.97)	1.02 (0.76–1.36)	0.90	1.13 (0.87–1.47)	0.36
	DP + SP (274)	197 (0.87)	0.92 (0.68–1.23)	0.56	1.05 (0.80–1.38)	0.71
Infant characteristics						
Sex	Male (441)	364 (1.00)	Reference group		Reference group	
	Female (414)	298 (0.87)	0.88 (0.69–1.12)	0.30	0.97 (0.78–1.21)	0.79
Sickle cell genotype	AA (668)	572 (1.03)	Reference group		Reference group	
	AS (181)	85 (0.57)	0.55 (0.40–0.76)	<0.001	0.61 (0.45–0.83)	0.001
	SS (6)	5 (1.05)	1.04 (0.26–4.26)	0.95	0.64 (0.19–2.18)	0.48
Born SGA	No (672)	510 (0.92)	Reference group		Reference group	
	Yes (183)	152 (1.00)	1.09 (0.81–1.46)	0.57	1.03 (0.79–1.34)	0.83
Date observation period began	Nov 2021–May 2023 (340)	471 (1.67)	Reference group		Reference group	
	Jun 2023–Oct 2023 (190)	101 (0.65)	0.39 (0.29–0.52)	<0.001	0.38 (0.29–0.51)	<0.001
	Nov 2023–Mar 2024 (325)	90 (0.33)	0.20 (0.15–0.26)	<0.001	0.20 (0.15–0.27)	<0.001

aIRR = adjusted incidence rate ratio; DP = dihydroartemisinin–piperaquine; IPTp = intermittent preventative treatment of malaria in pregnancy; IRR = incidence rate ratio; PPY = per person-year; SGA = small for gestational age; SP = sulfadoxine–pyrimethamine.

* Muiltivariate models are adjusted for all exposure variables assessed using univariate analyses.

### Malaria burden over the follow-up period.

Over the study period, there were 662 episodes of symptomatic malaria during 706.7 person-years of follow-up, corresponding to an overall incidence of 0.93 episodes per person-year (PPY). Incidence increased from 0.53 episodes PPY in children <3 months of age to 0.86 in children ≥3 months to <6 months of age and 1.05 episodes PPY in children ≥6 months to 12 months of age. The vast majority (650/662, 98.2%) of symptomatic malaria cases were uncomplicated. Among the 12 episodes of complicated malaria, five patients had only danger signs, six patients had severe anemia (hemoglobin <5 gm/dL), and one patient had jaundice. There were no deaths due to malaria. Considering all 662 malaria episodes, 231 (34.9%) occurred before 6 months of age, and 469 (70.8%) occurred before 9 months of age.

The overall prevalence of microscopic parasitemia at routine visits was 7.9%, whereas the combined prevalence of microscopic and submicroscopic parasitemia was 21.8%. Parasitemia increased progressively with infant age: microscopic prevalence rose from 5.2% at 8–16 weeks to 8.9% by 32–40 weeks, whereas microscopic or submicroscopic parasitemia rose from 13.6% to more than 25% across the same age ranges.

**Figure 2.**
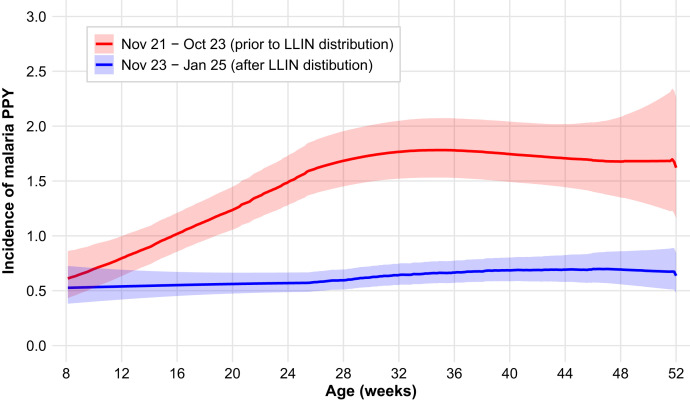
Incidence of malaria by age in weeks.

A marked decline in both malaria incidence and parasite prevalence was observed after the district-wide distribution of LLINs in October 2023. Malaria incidence dropped from 1.67 episodes PPY during the pre-intervention period (November 2021–October 2023) to 0.33 episodes PPY during the post-intervention period (November 2023–January 2025; Figure 2 and Supplemental Figure 1). The incidence of microscopic parasitemia fell from 12.8% between November 2021 and October 2023 to 6.9% between November 2023 and March 2024, and then fell to 2.6% between April 2024 and January 2025. Similar reductions were observed for microscopic or submicroscopic parasitemia (34.7–17.2% to 9.8%).

### Factors associated with the incidence of symptomatic malaria.

To better understand factors associated with the incidence of symptomatic malaria, associations were first examined in univariate models (Table 1). Infants residing in traditional housing had a 64% higher incidence of symptomatic malaria compared with those in modern housing (1.06 versus 0.65 episodes PPY; incidence rate ratio [IRR] = 1.64; 95% CI: 1.24–2.16; *P* <0.001) and those living in the poorest tertile of households had a 41% higher incidence compared with those in the least poor houses (1.07 versus 0.76 episodes PPY; IRR = 1.41; 95% CI: 1.04–1.91; *P* = 0.03). A similar trend was observed for maternal education, with a higher incidence among infants born to mothers with no or only primary education compared with those with secondary or higher education (1.00 versus 0.78 episodes PPY; IRR = 1.29; 95% CI: 0.99–1.68; *P* = 0.06). Infants with Hemoglobin AS (HbAS) had 45% fewer episodes of malaria compared with those with Haemoglobin AA (HbAA; 0.57 versus 1.03 episodes PPY; IRR = 0.55, 95% CI: 0.40–0.76; *P* <0.001). Regarding calendar time, incidence was highest among infants whose observation period began between November 2021 and May 2023 (1.67 PPY). It then decreased to 0.65 PPY in those whose observation period began between June 2023 and October 2023 (IRR = 0.39; *P* <0.001) and to 0.33 PPY in those whose observation period began between November 2023 and March 2024 (IRR = 0.20; *P* <0.001; Table 2).

**Table 2:** Risk factors for microscopic parasitemia at routine visits

Risk Factor	Category	Parasite Prevalence (%)	PR (95% CI)	*P*-Value	aPR (95% CI)	*P*-Value
Maternal and household characteristics						
Gravidity	Multigravid	574/7,715 (7.4)	Reference group		Reference group	
	Primigravid	206/2,251 (9.2)	1.22 (0.93–1.60)	0.15	1.22 (0.94–1.58)	0.14
Maternal education	Secondary or higher	204/3,009 (6.8)	Reference group		Reference group	
	None or primary	576/6,957 (8.3)	1.23 (0.93–1.63)	0.14	1.27 (0.97–1.66)	0.09
Type of house construction	Modern	131/2,945 (4.5)	Reference group		Reference group	
	Traditional	649/7,021 (9.2)	2.11 (1.53–2.91)	<0.001	1.92 (1.41–2.61)	<0.001
Household wealth categories	Least poor	183/3,185 (5.8)	Reference group		Reference group	
	Middle	304/3,483 (8.7)	1.51 (1.09–2.09)	0.01	1.21 (0.88–1.65)	0.24
	Poorest	293/3,298 (8.9)	1.55 (1.12–2.15)	0.009	1.18 (0.85–1.63)	0.33
Maternal IPTp regimen	SP	262/3,385 (7.7)	Reference group		Reference group	
	DP	279/3,408 (8.2)	1.07 (0.80–1.44)	0.65	1.12 (0.85–1.48)	0.43
	DP + SP	239/3,173 (7.5)	0.97 (0.72–1.31)	0.85	1.08 (0.81–1.45)	0.60
Infant characteristics						
Sex	Male	406/5,153 (7.9)	Reference group		Reference group	
	Female	374/4,813 (7.8)	0.98 (0.77–1.26)	0.90	1.06 (0.84–1.35)	0.61
Sickle cell genotype	AA	596/7,788 (7.7)	Reference group		Reference group	
	AS	180/2,111 (8.5)	1.10 (0.82–1.49)	0.51	1.14 (0.86–1.51)	0.36
	SS	4/67 (6.0)	0.83 (0.34–2.03)	0.69	0.45 (0.16–1.28)	0.13
Born SGA	No	569/7,832 (7.3)	Reference group		Reference group	
	Yes	211/2,134 (9.9)	1.37 (1.03–1.83)	0.03	1.26 (0.96–1.66)	0.10
Age at time of routine visit	8–16 weeks	133/2,537 (5.2)	Reference group		Reference group	
	20–28 weeks	213/2,515 (8.5)	1.61 (1.29–2.01)	<0.001	1.95 (1.55–2.44)	<0.001
	32–40 weeks	220/2,479 (8.9)	1.68 (1.33–2.13)	<0.001	2.39 (1.87–3.05)	<0.001
	44–52 weeks	214/2,435 (8.8)	1.67 (1.31–2.15)	<0.001	2.71 (2.09–3.51)	<0.001
Date of observation	Nov 2021–Oct 2023	536/4,174 (12.8)	Reference group		Reference group	
	Nov 2023–Mar 2024	148/2,145 (6.9)	0.73 (0.57–0.93)	0.01	0.64 (0.50–0.82)	<0.001
	Apr 2024–Jan 2025	96/3,647 (2.6)	0.38 (0.29–0.50)	<0.001	0.21 (0.16–0.29)	<0.001

aPR = adjusted prevalence ratio; DP = dihydroartemisinin–piperaquine; IPTp = intermittent preventative treatment of malaria in pregnancy; PR = prevalence ratio; SGA = small for gestational age; SP = sulfadoxine–pyrimethamine.

In multivariate models, both housing and maternal education remained independently associated with the incidence of symptomatic malaria. Infants in traditional housing had a 60% higher incidence of malaria (adjusted incidence rate ratio [aIRR] = 1.60; 95% CI: 1.23–2.08; *P* <0.001), and infants born to mothers with no or only primary education had a 34% higher incidence compared with those whose mothers attained at least secondary education (aIRR = 1.34; 95% CI: 1.05–1.71; *P* = 0.02). The protective effect of sickle cell trait persisted, with HbAS associated with a 39% lower incidence of malaria (aIRR = 0.61; 95% CI: 0.45–0.83; *P* = 0.001). The protective effect of calendar time in relation to universal LLIN distribution was also robust, with a 62% lower incidence in those whose observation period began between June 2023 and October 2023 and an 80% lower incidence in those whose observation period began between November 2023 and March 2024, compared with those whose observation period began between November 2021 and May 2023. Other factors, including maternal IPTp regimen in the DPSP study, gravidity, infant sex, household wealth, and being born SGA, were not independently associated with the incidence of symptomatic malaria (Table 1).

### Risk factors for parasitemia during routine visits.

Associations between exposures of interest and the risk of routine parasitemia largely mirrored those for symptomatic malaria, although there were some notable differences (Tables 2 and 3). In univariate analyses, the prevalence of microscopic parasitemia was significantly higher in children living in traditional housing compared with those living in modern housing (9.2% versus 4.5%; prevalence ratio [PR] = 2.11; 95% CI: 1.53–2.91; *P* <0.001), as well as those living in the poorest households compared with those living in the least poor households (8.9% versus 5.8%; PR = 1.55; 95% CI: 1.12–2.15; *P* = 0.009). Combined microscopic and submicroscopic parasitemia followed the same patterns (24.3% versus 15.8%; PR = 1.56; 95% CI: 1.29–1.90; *P* <0.001 and 24.3% versus 16.9%; PR = 1.45; 95% CI: 1.17–1.79; *P* = 0.001, respectively). Maternal education followed a similar trend, with a non-significantly higher prevalence among infants born to less-educated mothers (microscopic: PR = 1.23, *P* = 0.14; combined: PR = 1.18, *P* = 0.07).

**Table 3:** Risk factors for microscopic or submicroscopic parasitemia at routine visits

Risk Factor	Category	Parasite Prevalence (%)	PR (95% CI)	*P*-Value	aPR (95% CI)	*P*-Value
Maternal and household characteristics						
Gravidity	Multigravid	1,653/7,715 (21.4)	Reference group		Reference group	
	Primigravid	520/2,251 (23.1)	1.07 (0.88–1.29)	0.51	1.08 (0.92–1.27)	0.37
Maternal education	Secondary or higher	588/3,009 (19.5)	Reference group		Reference group	
	None or primary	1,585/6,957 (22.8)	1.18 (0.99–1.42)	0.07	1.18 (1.00–1.38)	0.05
Type of house construction	Modern	466/2,945 (15.8)	Reference group		Reference group	
	Traditional	1,707/7,021 (24.3)	1.56 (1.29–1.90)	<0.001	1.47 (1.23–1.76)	<0.001
Household wealth categories	Least poor	538/3,185 (16.9)	Reference group		Reference group	
	Middle	835/3,483 (24.0)	1.41 (1.15–1.74)	0.001	1.18 (0.98–1.43)	0.09
	Poorest	800/3,298 (24.3)	1.45 (1.17–1.79)	0.001	1.18 (0.97–1.43)	0.10
Maternal IPTp regimen	SP	733/3,385 (21.7)	Reference group		Reference group	
	DP	771/3,408 (22.6)	1.05 (0.86–1.28)	0.62	1.07 (0.90–1.27)	0.45
	DP + SP	669/3,173 (21.1)	0.97 (0.80–1.19)	0.79	1.03 (0.86–1.23)	0.73
Infant characteristics						
Sex	Male	1,128/5,153 (21.9)	Reference group		Reference group	
	Female	1,045/4,813 (21.7)	0.99 (0.84–1.17)	0.95	1.08 (0.94–1.24)	0.30
Sickle cell genotype	AA	1,724/7,788 (22.1)	Reference group		Reference group	
	AS	436/2,111 (20.7)	0.93 (0.75–1.15)	0.51	1.02 (0.85–1.23)	0.80
	SS	13/67 (19.4)	0.98 (0.45–2.11)	0.96	0.67 (0.35–1.28)	0.22
Born SGA	No	1,656/7,832 (21.1)	Reference group		Reference group	
	Yes	517/2,134 (24.2)	1.15 (0.95–1.39)	0.16	1.08 (0.91–1.27)	0.37
Age at time of routine visit	8–16 weeks	345/2,537 (13.6)	Reference group		Reference group	
	20–28 weeks	583/2,515 (23.2)	1.71 (1.50–1.94)	<0.001	2.00 (1.76–2.27)	<0.001
	32–40 weeks	623/2,479 (25.1)	1.85 (1.59–2.13)	<0.001	2.49 (2.15–2.88)	<0.001
	44–52 weeks	622/2,435 (25.5)	1.88 (1.62–2.19)	<0.001	2.80 (2.41–3.26)	<0.001
Date of observation	Nov 2021–Oct 2023	1,447/4,174 (34.7)	Reference group		Reference group	
	Nov 2023–Mar 2024	368/2,145 (17.2)	0.76 (0.65–0.88)	<0.001	0.64 (0.53–0.71)	<0.001
	Apr 2024–Jan 2025	358/3,647 (9.8)	0.56 (0.47–0.66)	<0.001	0.28 (0.23–0.34)	<0.001

aPR = adjusted prevalence ratio; DP = dihydroartemisinin–piperaquine; IPTp = intermittent preventative treatment of malaria in pregnancy; PR = prevalence ratio; SGA = small for gestational age; SP = sulfadoxine–pyrimethamine.

In multivariate analyses, housing and infant age remained independent predictors of parasitemia (Tables 2 and 3). Traditional housing was associated with a 92% higher risk of microscopic parasitemia (adjusted prevalence ratio [aPR] = 1.92; 95% CI: 1.41–2.61; *P* <0.001) and a 47% higher risk of combined microscopic or submicroscopic parasitemia (aPR = 1.47; 95% CI: 1.23–1.76; *P* <0.001). In infants aged 44–52 weeks, the risk of microscopic parasitemia (aPR = 2.71; 95% CI: 2.09–3.51; *P* <0.001) and the risk of combined microscopic or submicroscopic parasitemia (aPR = 2.80; 95% CI: 2.41–3.26; *P* <0.001) were nearly threefold higher compared with those of infants 8–16 weeks of age. Compared with the period between November 2021 and October 2023, the risk of microscopic parasitemia declined by 36% between November 2023 and March 2024 (aPR = 0.64; 95% CI: 0.50–0.82; *P* <0.001) and by 79% between April 2024 and January 2025 (aPR = 0.21; 95% CI: 0.16–0.29; *P* <0.001). Reductions in combined microscopic and submicroscopic parasitemia were similar (36% and 72%, respectively).

Interestingly, unlike its strong protective association with symptomatic malaria, sickle cell trait was not associated with reduced parasite prevalence in either the unadjusted or adjusted analyses. In the adjusted analyses, infants with HbAS had a slightly higher, although nonsignificant, risk of both microscopic parasitemia (aPR = 1.14; 95% CI: 0.86–1.51; *P* = 0.36) and combined microscopic and submicroscopic parasitemia (aPR = 1.02; 95% CI: 0.85–1.23; *P* = 0.80) compared with infants with HbAA. This contrast suggests that HbAS does not reduce the risk of infection with malaria parasites but rather confers protection against progression to symptomatic disease. Other covariates, including maternal IPTp regimen, gravidity, maternal education, household wealth, sex, and SGA status, were not independently associated with parasitemia in multivariate analyses.

## DISCUSSION

The present prospective cohort study provides contemporary evidence on the natural history and burden of malaria in infants during their first year of life in a high-transmission setting. Malaria represented a substantial health burden even in very young infants, with important implications for prevention strategies and clinical practice. The overall malaria incidence of nearly one episode per child per year observed in the study cohort underscores the significant disease burden in this vulnerable population.

Limited literature exists on malaria prevention and management in young infants living in perennial high-transmission settings, contributing to the continued lack of attention to this risk group in research and policy.^7^^,^^15^^,^^16^ Although the WHO recommends PMC with SP in settings with perennial transmission, it is not currently used in Uganda, where there are very high rates of SP resistance,^17^ or in most other countries in sub-Saharan Africa. Malaria vaccination is being widely rolled out in Uganda and across Africa this year; however, standard vaccine dosing begins at 5–6 months of age, and optimal vaccine efficacy is achieved after only three doses (administered at 9 months of age in accordance with Uganda dosing guidelines). In the present study, 35% of malaria episodes during the first year of life occurred before 6 months of age, and 71% occurred before 9 months of age. Although most malaria cases in the study cohort were uncomplicated, participants were encouraged to visit a dedicated study clinic, open 7 days per week, for all medical care and were reimbursed for transportation. The low rates of severe malaria and the absence of malaria-related deaths in the study cohort can be attributed to close follow-up, the timely identification of malaria cases, and the prompt and effective treatment of these cases. In a real-world setting, where access to prompt and effective care may be lacking, the burden of severe disease would likely be higher, especially in the youngest children, due to immature immunity.^18^ There is an urgent need for the development and testing of age-appropriate preventive interventions targeting young infants, such as PMC with DP or other drugs, malaria vaccines administered at earlier ages, the administration of protective monoclonal antibodies,^19^ and permethrin-treated baby wraps.^20^ Furthermore, treatment and chemoprevention guidelines often lack clear diagnostic and dosing recommendations for young infants and those who weigh less than 5 kg.^21^ This indicates a need for tailored diagnostic protocols and drug formulations that are palatable and weight-specific to this age group. Addressing these gaps will be essential for reducing early childhood morbidity and mortality from malaria.

Sickle cell trait was relatively common in this high-transmission setting, consistent with published estimates from Uganda,^22^ and the differential effects of sickle cell trait on symptomatic malaria versus parasitemia provide insights into the mechanisms of protection. Although HbAS conferred significant protection against symptomatic malaria (39% lower incidence), consistent with previous studies,^8^^,^^23^ it exhibited no protective effect against parasitemia (either microscopic or submicroscopic). This pattern suggests that sickle cell trait does not decrease the risk of initial infection but rather protects against progression to clinical disease, consistent with proposed mechanisms that involve reduced parasite multiplication or enhanced immune recognition of infected cells.^24^^,^^25^

The dramatic reduction in malaria incidence after the district-wide distribution of LLINs containing alphacypermethrin plus chlorfenapyr in October 2023 strongly suggests that next-generation, dual-active-ingredient bednets can improve protection of infants. The 80% reduction in incidence (from 1.67 to 0.33 episodes PPY) revealed that LLINs treated with alphacypermethrin plus chlorfenapyr can provide substantial protection, even in areas with pyrethroid-resistant mosquitoes. This finding supports WHO recommendations for the use of next-generation LLINs in areas with confirmed pyrethroid resistance. The parallel reductions in both microscopic and submicroscopic parasitemia prevalence after LLIN distribution suggest that the intervention reduced malaria transmission and disease.^26^^,^^27^ This finding has broad implications for community-level malaria control because reduced transmission can benefit both protected and unprotected individuals through indirect effects.^28^ In addition, these results emphasize the importance of maintaining regular distribution campaigns and ensuring proper and consistent net usage at the household level to sustain and further gains in malaria control and in preventing malaria in infancy.

The study findings also highlight the persistent influence of socioeconomic factors on malaria risk in infants. Infants who reside in traditionally constructed houses—typically characterized by mud walls, thatched roofs, and open eaves—are at a higher risk of exposure to malaria vectors.^29^^,^^30^ Traditional housing construction was the strongest predictor of both symptomatic malaria and parasitemia, with infants in traditional houses experiencing 60% higher incidence of clinical episodes and nearly twice the risk of microscopic parasitemia. In contrast, improved housing structures with sealed walls and screened windows offer a protective barrier. The association between maternal education and malaria risk, although modest, underscores the importance of health literacy and decision-making capacity in malaria prevention. Mothers with higher education may be more likely to adopt preventive measures, such as consistently using insecticide-treated nets^31^ and seeking prompt medical care, thereby reducing malaria infection rates.^31^^,^^32^

Interestingly, in the present study, maternal characteristics associated with protection against malaria in pregnancy (including gravidity and the receipt of more effective IPTp regimens) were not associated with malaria risk in infants. This contrasts with results from several observational studies, which have suggested that malaria during pregnancy, particularly placental malaria, may increase the risk of infant malaria.^33^^–^^35^ However, this association is likely confounded by shared exposure risks between mother and infant. The study data suggest that mechanisms of protection during pregnancy may not impact infant outcomes.

The present study has several strengths, including its large sample size, prospective design, high level of retention, and use of both microscopy and qPCR to measure malaria burden. However, there were some limitations. First, the study was conducted in a single geographic region with specific transmission dynamics and healthcare practices, which may limit the generalizability of the findings to other malaria-endemic settings with different environmental, socioeconomic, or healthcare factors. Second, although the decline in malaria prevalence after LLIN distribution supports the effectiveness of this intervention, the present study lacked detailed individual-level data on net use and maintenance, which would have strengthened our causal inference. Third, the number of infants with sickle cell disease was small, limiting the power to study its association with malaria outcomes. Fourth, because of the study design, infants were enrolled at 4–8 weeks of age. Therefore, results were not obtained on the malaria burden in children younger than this age group, although it is known that very little malaria occurs before 4 weeks of age.

## CONCLUSION

The present study revealed that malaria represents a substantial and underappreciated burden in infants during their first year of life in high-transmission settings. The early onset of susceptibility, marked biological and social determinants of risk, and the dramatic impact of vector control interventions have important implications for prevention strategies and clinical care. Addressing this burden will require expanded prevention strategies targeting younger infants, continued investment in effective vector control, and attention to the social determinants that perpetuate malaria risk in the most vulnerable populations. The evidence presented here should inform policy discussions about extending malaria prevention strategies to cover the critical first months of life and guide clinical practice toward greater recognition of malaria risk in young infants.

## Supplemental Materials

10.4269/ajtmh.25-0695 Supplemental Materials

## References

[1] World Health Organization, 2024. *World Malaria Report, 2024*. Available at: https://www.who.int/teams/global-malaria-programme/reports/world-malaria-report-2024. Accessed March 9, 2026.

[2] D’Alessandro U, , 2012. Malaria in infants aged less than six months – Is it an area of unmet medical need? Malar J 11: 400. 23198986 10.1186/1475-2875-11-400 PMC3529680

[3] Kitua AY Smith T Alonso PL Masanja H Urassa H Menendez C Kimario J Tanner M, 1996. *Plasmodium falciparum* malaria in the first year of life in an area of intense and perennial transmission. Trop Med Int Health 1: 475–484. 8765455 10.1046/j.1365-3156.1996.d01-89.x

[4] Natama HM, , 2023. Associations between prenatal malaria exposure, maternal antibodies at birth, and malaria susceptibility during the first year of life in Burkina Faso. Infect Immun 91: e0026823. 37754682 10.1128/iai.00268-23 PMC10580994

[5] Menendez C, , 2007. Varying efficacy of intermittent preventive treatment for malaria in infants in two similar trials: Public health implications. Malar J 6: 132. 17897454 10.1186/1475-2875-6-132 PMC2100068

[6] Dobbs KR Dent AE, 2016. *Plasmodium* malaria and antimalarial antibodies in the first year of life. Parasitology 143: 129–138. 26743626 10.1017/S0031182015001626 PMC4825094

[7] Larru B Molyneux E Ter Kuile FO Taylor T Molyneux M Terlouw DJ, 2009. Malaria in infants below six months of age: Retrospective surveillance of hospital admission records in Blantyre, Malawi. Malar J 8: 310. 20038299 10.1186/1475-2875-8-310 PMC2805692

[8] Zehner N, , 2021. Age-related changes in malaria clinical phenotypes during infancy are modified by sickle cell trait. Clin Infect Dis 73: 1887–1895. 33738485 10.1093/cid/ciab245 PMC8599196

[9] Aponte JJ, , 2009. Efficacy and safety of intermittent preventive treatment with sulfadoxine-pyrimethamine for malaria in African infants: A pooled analysis of six randomised, placebo-controlled trials. Lancet 374: 1533–1542. 19765816 10.1016/S0140-6736(09)61258-7

[10] Gesase S, , 2009. High resistance of *Plasmodium falciparum* to sulphadoxine/pyrimethamine in northern Tanzania and the emergence of dhps resistance mutation at Codon 581. PLoS One 4: e4569. 19238219 10.1371/journal.pone.0004569 PMC2644264

[11] Kakuru A, , 2025. Dihydroartemisinin–piperaquine plus sulfadoxine–pyrimethamine versus either drug alone for intermittent preventive treatment of malaria in pregnancy: A double-blind, randomized, controlled trial. PLoS Med 22: e1004582. 40966190 10.1371/journal.pmed.1004582 PMC12445490

[12] Republic of Uganda Ministry of Health, 2023. *Uganda Clinical Guidelines 2023: National Guidelines for Management of Common Health Conditions*. Available at: https://www.differentiatedservicedelivery.org/wp-content/uploads/UCG-2023-Publication-Final-PDF-Version-1.pdf. Accessed March 9, 2026.

[13] Hofmann N Mwingira F Shekalaghe S Robinson LJ Mueller I Felger I, 2015. Ultra-sensitive detection of *Plasmodium falciparum* by amplification of multi-copy subtelomeric targets. PLoS Med 12: e1001788. 25734259 10.1371/journal.pmed.1001788 PMC4348198

[14] Okiring J Olwoch P Kakuru A Okou J Ochokoru H Ochieng TA Kajubi R Kamya MR Dorsey G Tusting LS, 2019. Household and maternal risk factors for malaria in pregnancy in a highly endemic area of Uganda: A prospective cohort study. Malar J 18: 144. 31014336 10.1186/s12936-019-2779-x PMC6480498

[15] Kalkman LC Hanscheid T Krishna S Kremsner PG Grobusch MP, 2022. Antimalarial treatment in infants. Expert Opin Pharmacother 23: 1711–1726. 36174125 10.1080/14656566.2022.2130687

[16] Mbonye MK Burnett SM Naikoba S Colebunders R Wouters K Weaver MR Van Geertruyden JP, 2015. Malaria care in infants aged under six months in Uganda: An area of unmet needs! PLoS One 10: e0123283. 25860016 10.1371/journal.pone.0123283 PMC4393291

[17] Okitwi M, , 2025. Changes in susceptibility of *Plasmodium falciparum* to antimalarial drugs in Uganda over time: 2019–2024. Nat Commun 16: 7353. 40783405 10.1038/s41467-025-62810-x PMC12335539

[18] Callaway PC Farrington LA Feeney ME, 2021. Malaria and early life immunity: Competence in context. Front Immunol 12: 634749. 33679787 10.3389/fimmu.2021.634749 PMC7933008

[19] Kayentao K, , 2022. Safety and efficacy of a monoclonal antibody against malaria in Mali. N Engl J Med 387: 1833–1842. 36317783 10.1056/NEJMoa2206966 PMC9881676

[20] Boyce RM, , 2025. Permethrin-treated baby wraps for the prevention of malaria. N Engl J Med 393: 1399–1408. 40991921 10.1056/NEJMoa2501628 PMC12462887

[21] World Health Organization, 2025. *WHO Guidelines for Malaria*. Available at: https://www.who.int/publications/i/item/guidelines-for-malaria. Accessed March 9, 2026.

[22] Ndeezi G, , 2016. Burden of sickle cell trait and disease in the Uganda Sickle Surveillance Study (US3): A cross-sectional study. *Lancet Glob Health* 4: e195–e200. 26833239 10.1016/S2214-109X(15)00288-0

[23] Williams TN Mwangi TW Wambua S Alexander ND Kortok M Snow RW Marsh K, 2005. Sickle cell trait and the risk of *Plasmodium falciparum* malaria and other childhood diseases. J Infect Dis 192: 178–186. 15942909 10.1086/430744 PMC3545189

[24] Williams TN Mwangi TW Roberts DJ Alexander ND Weatherall DJ Wambua S Kortok M Snow RW Marsh K, 2005. An immune basis for malaria protection by the sickle cell trait. PLoS Med 2: e128. 15916466 10.1371/journal.pmed.0020128 PMC1140945

[25] Gong L Maiteki-Sebuguzi C Rosenthal PJ Hubbard AE Drakeley CJ Dorsey G Greenhouse B, 2012. Evidence for both innate and acquired mechanisms of protection from *Plasmodium falciparum* in children with sickle cell trait. Blood 119: 3808–3814. 22327223 10.1182/blood-2011-08-371062 PMC3335384

[26] Kleinschmidt I, , 2018. Implications of insecticide resistance for malaria vector control with long-lasting insecticidal nets: A WHO-coordinated, prospective, international, observational cohort study. Lancet Infect Dis 18: 640–649. 29650424 10.1016/S1473-3099(18)30172-5 PMC5968369

[27] Protopopoff N, , 2018. Effectiveness of a long-lasting piperonyl butoxide-treated insecticidal net and indoor residual spray interventions, separately and together, against malaria transmitted by pyrethroid-resistant mosquitoes: A cluster, randomised controlled, two-by-two factorial design trial. Lancet 391: 1577–1588. 29655496 10.1016/S0140-6736(18)30427-6 PMC5910376

[28] Benjamin-Chung J, , 2024. Extension of efficacy range for targeted malaria-elimination interventions due to spillover effects. Nat Med 30: 2813–2820. 38965434 10.1038/s41591-024-03134-z PMC11483210

[29] Coleman M Coleman M Mabaso MLH Mabuza AM Kok G Coetzee M Durrheim DN, 2010. Household and microeconomic factors associated with malaria in Mpumalanga, South Africa. Trans R Soc Trop Med Hyg 104: 143–147. 19732924 10.1016/j.trstmh.2009.07.010

[30] Habermann T Wafula ST May J Lorenz E Puradiredja DI, 2024. The mediating role of behavioural and socio-structural factors on the association between household wealth and childhood malaria in Ghana. Malar J 23: 370. 39673072 10.1186/s12936-024-05204-6 PMC11645786

[31] Siri JG, 2014. Independent associations of maternal education and household wealth with malaria risk in children. *Ecol Society* 19: 33.

[32] Njau JD Stephenson R Menon MP Kachur SP McFarland DA, 2014. Investigating the important correlates of maternal education and childhood malaria infections. Am J Trop Med Hyg 91: 509–519. 25002302 10.4269/ajtmh.13-0713 PMC4155551

[33] Bardají A Sigauque B Sanz S Maixenchs M Ordi J Aponte JJ Mabunda S Alonso PL Menéndez C, 2011. Impact of malaria at the end of pregnancy on infant mortality and morbidity. J Infect Dis 203: 691–699. 21199881 10.1093/infdis/jiq049 PMC3071276

[34] Le Port A, , 2011. Infections in infants during the first 12 months of life: Role of placental malaria and environmental factors. PLoS One 6: e27516. 22096588 10.1371/journal.pone.0027516 PMC3214070

[35] Barry A, , 2025. Preterm birth and malaria susceptibility in offspring of uninfected multigravid women. JAMA Netw Open 8: e2532179. 40956579 10.1001/jamanetworkopen.2025.32179 PMC12441872

