# Supplementary figures and images for: Natural History and the Burden of Malaria During the First Year of Life in the High-Transmission Setting of Uganda
Source: Am J Trop Med Hyg. 2026 Apr 2;114(5):905–12. doi: 10.4269/ajtmh.25-0695 (PMC13153588; doi:10.4269/ajtmh.25-0695)

**Supplemental Figure 1.** Incidence of malaria by age in weeks.

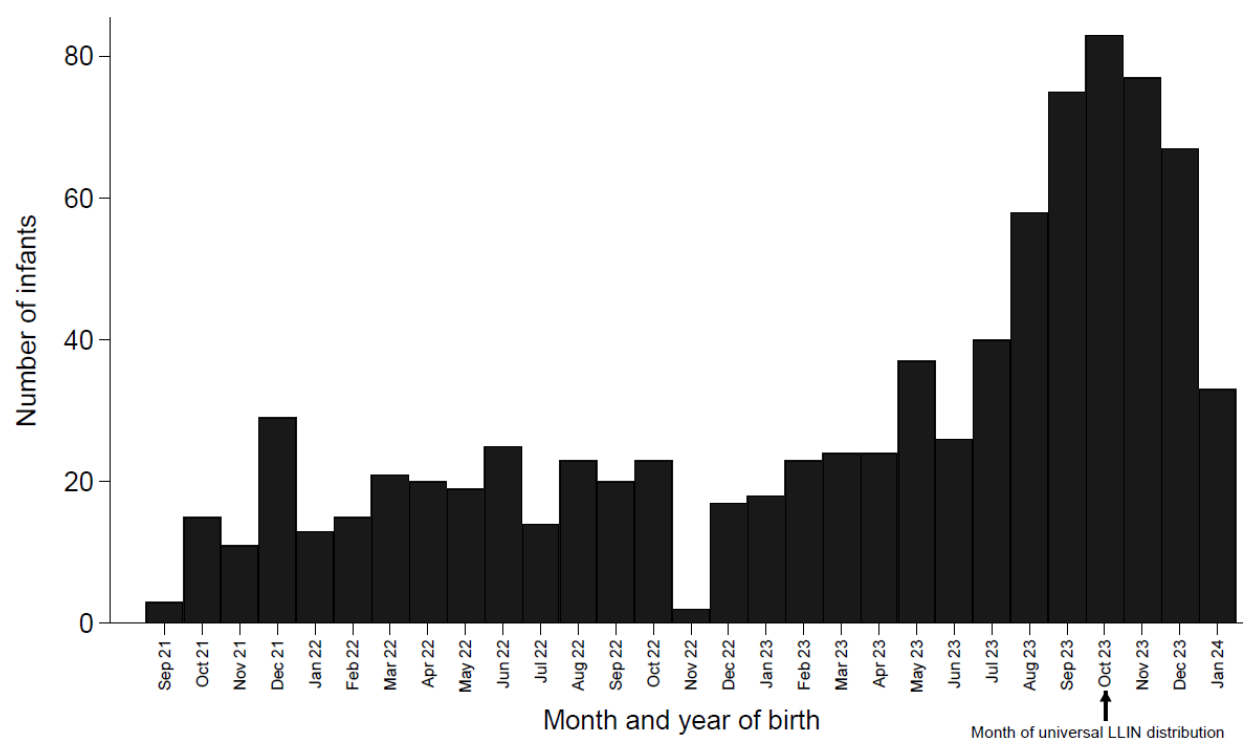

Supplement: Supplemental Materials [file tpmd250695.SD1.pdf]
